# The production of ‘food boluses’ by Antarctic krill and implications for organic matter transport

**DOI:** 10.1098/rsbl.2025.0312

**Published:** 2025-10-08

**Authors:** Anita Butterley, So Kawaguchi, Lennart Thomas Bach, Kerrie Swadling

**Affiliations:** ^1^ University of Tasmania Institute for Marine and Antarctic Studies, Hobart, Tasmania, Australia; ^2^ Department of the Environment, Water, Heritage and the Arts, Australian Antarctic Division, Kingston, Tasmania, Australia

**Keywords:** *Euphausia superba*, filter feeding, feeding behaviour, carbon flux

## Abstract

Antarctic krill (*Euphausia superba*) are a key species in the marine Antarctic ecosystem. Food boluses, a by-product of feeding where Antarctic krill form a compact food mass within their feeding basket, were formed and rejected under laboratory conditions. We explored the conditions leading to bolus formation by examining feeding behaviour of Antarctic krill in response to different phytoplankton types and concentrations. Two scenarios were observed that increased the likelihood of bolus formation: (i) when food concentrations exceed the krill’s filtering capacity and (ii) when particles are caught in the feeding basket. We measured the frequency of rejection of boluses, along with their composition, carbon and nitrogen contents, and sinking rates. For cell concentrations approximately 10^8^ cells l^−1^, the frequency of rejection ranged from 2.6 to 17.0 boluses per hour. The carbon and nitrogen contents averaged 24.1 µg C mm^−3^ and 2.3 µg N mm^−3^, and sinking rates averaged 367 m d^−1^. Our findings suggest this behaviour may also occur *in situ* and could contribute to organic carbon export, with bolus sinking rates comparable to or exceeding those of Antarctic krill faecal pellets. If confirmed in the field, this behaviour may also occur in other krill species with similar feeding behaviours.

## 1. Introduction

Antarctic krill (*Euphausia superba*; hereafter krill) are an integral part of the Southern Ocean ecosystem, linking primary producers to higher trophic levels and comprising the largest biomass of any species [1,2]. This is due, in part, to their ability to feed on phytoplankton concentrations as low as 0.5 µg chlorophyll *a* l^−1^ while still reaching considerable growth rates [3]. Krill feed in open waters, under sea-ice and at depth [3,4], resulting in diverse feeding behaviours. Krill have varied diets, consuming phytoplankton as their main food source, along with zooplankton and detritus [3,5,6].

High feeding efficiency is enabled by the complex nature of their filtering apparatus, known as the feeding basket (electronic supplementary material, figure S1). The six pairs of thoracopods (electronic supplementary material, figure S1) make up a three-dimensional structure with three levels of setae, forming a mesh size of 2−3 µm [7]. This allows the capture of small and large particles, ranging from 2 µm to over 2 mm [8]. Antarctic krill primarily capture particles via filter feeding, with swarms of krill grazing down phytoplankton blooms in a matter of hours. Phytoplankton concentrations in the open Southern Ocean typically reach up to 1 × 10⁷ cells l^−1^ during productive periods, while dense coastal blooms may approach 10⁸ cells l^−1^ [9,10]. Krill are capable of feeding efficiently across a range of concentrations, but their behaviour may shift under food-saturated conditions, such as during coastal blooms, where their filtering and ingestion capacity can be exceeded [3,11].

The process of filter feeding in Antarctic krill is well established [7,12,13]. During this process, a food bolus can form within the feeding basket, as particles stick together to form a compact mass, which is often ejected from the basket. This behaviour by Antarctic krill is yet to be studied in detail, although boluses were first mentioned in 1983 when an *in vitro* experiment fed Antarctic krill with high algae concentrations [14]. And in 1986, boluses were found within the feeding baskets of other euphausiids, namely *Thysanoessa raschi* and *Meganyctiphanes norvegica, in situ* [15]. These were the first observations of food boluses being produced by krill, but no further investigations were made into the conditions or external factors influencing their formation.

This study addresses this knowledge gap using laboratory-based feeding experiments with Antarctic krill. Our main objective was to identify behaviours associated with bolus formation and their structural make-up. Two major phytoplankton groups were used: flagellates and diatoms. Flagellates and the diatom *Phaeodactylum tricornutum* were selected as they were standard diets in the Australian Antarctic Division (AAD) krill aquarium, with additional Antarctic diatoms included for broader comparisons. An additional aim was to evaluate whether boluses have the potential to contribute to vertical organic matter flux, thereby influencing carbon export processes in the Southern Ocean.

## 2. Material and methods

### (a) Krill aquaria

Food boluses were collected from krill held in the AAD aquaria in Tasmania [16]. These krill were captured during the 2017/2018 summer in the Indian Sector of the Southern Ocean and had been in captivity for approximately 2.5 years at the time of the experiment in 2021, with an estimated age of 4−7 years.

### (b) Culturing phytoplankton

Phytoplankton cultures of the diatoms *Proboscia inermis*, *Chaetoceros flexuousus* and *Fragilariopsis cylindrus* were grown in 1 l bottles containing filtered seawater enriched with *f/2* media and silicate. Cultures were maintained at 2.5°C under constant light (30 µmol photons m² s^−1^). The flagellates *Geminigera cryophila* and *Pyramimonas gelidicola*, along with the diatom *P. tricornutum,* were maintained long term in 120 l bags under routine culturing conditions at AAD [17].

### (c) High resolution observations of bolus formation

A Leica DFC 310 FX camera was used to film individual krill tethered with a nylon noose around the tail, which allowed for easy release after experiments (electronic supplementary material, video S1). The individual krill was placed in a 50 × 29 × 2.9 cm flow-through chamber filled with 0.5°C filtered seawater. The system generated a water flow of 10 cm s^−1^, approximating the natural swimming speed of feeding krill [14]. While tethered individuals showed fewer filter-feeding bouts compared to free-swimming individuals, they still displayed the full behavioural sequence associated with bolus formation.

Phytoplankton mixtures (*G. cryophila*, *P. tricornutum* and *P. gelidicola*) were introduced upstream to observe the processes leading to bolus formation. For detailed mouthpart analysis, individual krill were placed in a smaller chamber (5 × 2 × 2 cm) with a viewing window submerged in a near-frozen water bath to maintain temperatures below 1°C. These observations were conducted using a Leica M205C stereomicroscope.

### (d) Collection and visual analysis of boluses

Krill were starved for 24 h before all experiments. Six krill per bucket (4–5 cm standard length) were introduced into 15 l buckets placed in a 0.5°C water bath. The buckets contained a grate with 1 cm square openings at the bottom to separate krill from boluses and faecal pellets. Monoculture diets of *G. cryophila*, *P. gelidicola* and *P. tricornutum* were provided in excess. Due to culturing limitations, *C. flexuousus*, *P. inermis* and *F. cylindrus* were used in limited experiments. Boluses and faecal pellets (electronic supplementary material, figure S2) were collected using a glass pipette and examined within 30 min under a Leica M205C stereomicroscope (40× magnification). The diameter of boluses was measured using ImageJ software [18]. Volumes were calculated assuming boluses were ellipsoids:


$$Bolus^{\prime }\;volume=\left(\frac{4}{3}\right)\times \pi \times (diameter\times radius^{2})$$

Cells within boluses were imaged at 400× magnification using an Axioskop compound microscope and Canon EOS 5D camera looking for cell fragmentation during bolus formation.

One of the initial experiments was contaminated by blue plastic. Therefore, contamination controls were implemented, including the use of clean equipment and covered containers, and if any experiments were found to be contaminated, the results were discarded and the experiment repeated.

### (e) Sinking rate measurements and elemental composition

Sinking velocities were measured in a temperature-controlled room (2°C) using boluses and faecal pellets collected as above. Measuring cylinders (50 × 6.5 cm) were left to equilibrate for ≥ 24 h before measurements to eliminate thermal gradients and minimize convection. Boluses from *G. cryophila* (*n* = 52), *P. gelidicola* (*n* = 54) and *P. tricornutum* (*n* = 40) were carefully introduced, and sinking rates recorded over a 10 cm vertical section once sinking stabilized [19]. Faecal pellets produced on the same diets were measured similarly (*n* = 44, *n* = 38 and *n* = 27, respectively). Elemental carbon and nitrogen content of 1−3 boluses per treatment was analysed using a Thermo Finnigan EA 1112 Series Flash Elemental Analyser [20].

### (f) Quantification of bolus production and rejection after 5 h

Bolus production was conducted in 8 l buckets with grated bottoms, filled with seawater maintained at −1°C. Phytoplankton were added at increasing concentrations: 2.5 × 10⁷, 5 × 10⁷ and 10⁸ cells l^−1^. After 24 h of starvation, five krill (3–5 cm standard length) were introduced to each bucket, and boluses and faecal pellets were counted after 5 h.

Bolus presence was also monitored to assess potential interference with feeding. The filtering activity was evaluated by observing the lateral spread of thoracopods and comparing the number of compressions recorded using a Canon Powershot SX60 camera (electronic supplementary material, video S2).

## 3. Results

### (a) Feeding behaviour and bolus formation

This study analysed how external food conditions, specifically the type and concentration of phytoplankton, influenced bolus formation in adult krill when food was abundant (i.e. when phytoplankton concentrations were enough to exceed the krill’s filtering and ingestion capacity). Tethered krill were observed beating their thoracopods rapidly after phytoplankton were added. Compared with untethered krill, the thoracopods of tethered krill did not fan out and extend as far laterally (especially legs 5 and 6) resulting in bouts of up to 6 beats per second, twice as much as untethered krill. Although movement was limited, boluses were still formed (electronic supplementary material, video S1).

**Figure 1.**
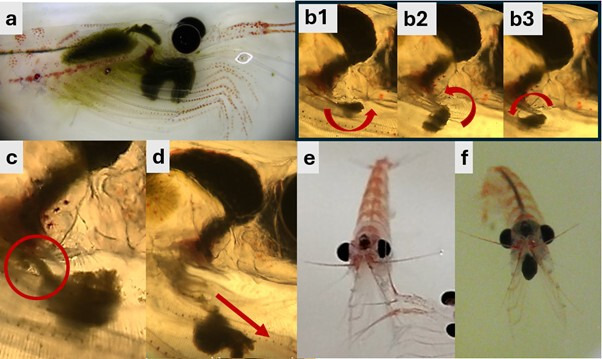
Krill forming boluses with (a) the position of a bolus in the feeding basket of krill, (b1−3) bolus being rotated by appendages with arrows showing anticlockwise direction, (c) bolus being pulled down to form an ingestible strand, (d) frontal thoracopods removing bolus from feeding basket in direction of arrow, (e) the lateral spread of thoracopods during filter feeding (note: a second krill appears in the bottom right) and (f) their lateral spread while a bolus is present in the feeding basket.

Boluses initially formed at the mouth, growing in size between the ischia (figure 1a; electronic supplementary material, figure S2). Phytoplanktons were caught in the feeding basket and moved toward the thoracic groove then moved towards the mandibular palps, where grinding and tearing of particles began (electronic supplementary material, video S3). By this stage, a matrix had formed binding the bolus. Prehensile appendages manipulated the growing bolus by occasionally rotating it anticlockwise (figure 1b) and pulling it away from the mouth to make a strand for ingestion (figure 1c). Once the bolus exceeded the size of the extended mandibles, it could no longer be rolled, though particles continued to accumulate. The mandibles continued to pull the bolus away from the mouthparts to continue making the strand for ingestion, which also attached the bolus to the individual. When the individual became agitated, the prehensile appendages pushed the bolus away from the mouth and the thoracic limbs thrust it out of the basket (figure 1d). It was noted in this case that dislodging the bolus from the basket was most probably due to stress caused by holding on to a bolus that had grown larger than their capacity to handle it. The rate of thoracopod compressions remained unchanged with or without a bolus present, indicating that filter-feeding was not affected (figure 1e,f).

Free-swimming krill began filter feeding upon the introduction of phytoplankton. Boluses formed within 5 min at high food concentrations (electronic supplementary material, video S2), and krill continued feeding for up to 2 h. Bolus rejection in the 5 h period varied with diet and food concentration. At 2.5 × 10^7^ cells l^−1^, only one bolus was rejected, while at 5 × 10^7^ cells l^−1^, 21 boluses were rejected. At 10^8^ cells l^−1^, rejection rates differed by diet, with *P. tricornutum* causing the highest rejection (approx. 139 boluses), while *G. cryophila* and *P. gelidicola* led to fewer rejections (approx. 28 and 31, respectively) (electronic supplementary material, table S1).

### (b) Structure and visual characteristics

**Figure 2.**
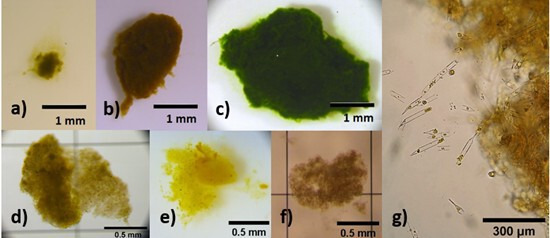
Boluses produced from various diets. (a) *P. tricornutum*, (b) *G. cryophila,* (c) *P. gelidicola,* (d) *C. flexuousus,* (e) *P. inermis,* (f) *F. cylindrus* and (g) edge of *P. inermis* bolus showing cell fragmentation.

Bolus structure and appearance varied with diet and food concentration. Generally, boluses resembled flattened spheres, compressed within the feeding basket (figure 1f). Diatom boluses were rejected quickly and appeared fluffy and porous, whereas flagellate boluses were dense and more compact (figure 2). At 400× magnification, boluses showed fragmented cells (figure 2g).

Krill fed *C. flexuousus*, *P. inermis* and *F. cylindrus* at intermediate concentrations (5 × 10^7^ cells l^−1^) rejected boluses rapidly, with 6−8 ejected within 10 min. These boluses had a mean volume of 0.1 mm^3^ and were visually similar to *P. tricornutum* boluses, appearing fluffy and less dense than flagellate boluses (figure 2d–f).

**Figure 3.**
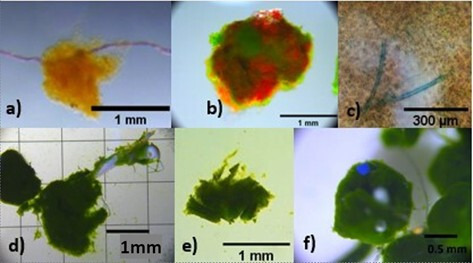
Artificial and biological products found in boluses. (a) Thread, (b) red wool, (c) stained cyanobacteria, (d) krill pieces, (e) krill faecal pellet and (f) plastics.

Microscopy revealed biological and artificial particles within boluses, including setae, faecal pellets, nematodes, krill flesh, threads and plastics, ranging from 0.1 to 9 mm in length (figure 3). Contaminated boluses were excluded from further analysis.

### (c) Sinking velocities and sizes

Sinking velocities of boluses ranged from 42 to 1403 m d^−1^. Boluses from *P. tricornutum*, *G. cryophila* and *P. gelidicola* showed mean sinking rates of 140 ± 103, 321 ± 212 and 568 ± 293 m d^−1^, respectively. *Pyramimonas gelidicola* boluses sank the fastest, as did faecal pellets from the same diet. While *P. tricornutum* boluses had the slowest sinking speeds, their faecal pellets sank faster than those from *G. cryophila*. Overall, faecal pellets sank more slowly than boluses, with the fastest pellets reaching 468 m d^−1^ compared with 1403 m d^−1^. The mean sinking velocity for faecal pellets was 269 m d^−1^, while boluses averaged 367 m d^−1^.

Bolus size varied by diet, with a mean diameter of 1.43 mm. *P. gelidicola* produced the largest boluses (1.73 ± 0.92 mm), followed by *G. cryophila* (1.59 ± 0.99 mm). Both were significantly larger than boluses from *P. tricornutum* (0.93 ± 1.07 mm; electronic supplementary material, table S2).

### (d) Elemental analysis

Mean carbon and nitrogen contents of boluses were 24.0 µg C mm^−3^ and 2.3 µg N mm^−3^ (electronic supplementary material, Table S3). Carbon content varied by diet, with boluses from *P. tricornutum* containing 17.0 ± 3.0 µg C mm^−3^, *G. cryophila* had 19.9 ± 1.9 µg C mm^−3^ and *P. gelidicola* with 35.4 ± 8.9 µg C mm^−3^. Nitrogen content was similar between *P. tricornutum* (1.8 ± 0.1 µg N mm^−3^) and *G. cryophila* (1.9 ± 0.2 µg N mm^−3^), with a slightly elevated value for *P. gelidicola* (3.2 ± 0.8 µg N mm^−3^). C : N ratios were broadly similar across diets, averaging approximately 12. One result from *P. tricornutum* was discarded due to contamination, resulting in a sample size of three.

## 4. Discussion

Here, we provide the first detailed observations of bolus formation and subsequent rejection by krill feeding on multiple phytoplankton genera across varying concentrations. Our results indicate that krill exhibit efficient feeding strategies under food-saturated conditions, with bolus formation becoming more frequent at concentrations exceeding 2.5 × 10^7^ cells l^−1^. While this exceeds typical background concentrations in the open Southern Ocean (i.e. 1 × 10^7^ cells l^−1^) [10], it is within the bounds of coastal phytoplankton blooms, which can approach 10^8^ cells l^−1^ [9]. We note that 2.5 × 10⁷ cells l^−1^ was the lowest concentration tested in this study and while boluses were observed at this level, we cannot determine the minimum threshold concentration required for bolus formation, and further work at lower food concentrations would be needed to resolve this. At 2.5 × 10^7^ cells l^−1^, krill were observed steadily filtering and consuming phytoplankton at up to four compressions of the feeding basket per second. At this concentration, food boluses were rarely formed and rejected, with only one bolus being rejected across all phytoplankton groups during the 5 h period.

Bolus formation in krill has been linked to satiation, with previous studies reporting a pause in feeding until the bolus was ingested or rejected, and feeding stopping once satiated [13]. In contrast, our study found boluses were not ingested once visible in the basket. Rather, boluses were either continually eaten as the krill continued to filter the surrounding water or rejected by the krill before they continued to feed at the previous rate. Krill also continued to swim while still adding to their bolus once their digestive gland was full rather than sinking as previously described [11,14].

Although boluses were observed at all tested concentrations (2.5 × 10^7^, 5 × 10^7^ and 10^8^ cells l^⁻¹^), the formation and rejection rates rose sharply at high phytoplankton concentrations (approx. 10^8^ cells l^⁻¹^), with 28−139 rejected depending on the diet*.* Krill fed voraciously in these conditions, continually filtering even when their basket became saturated with phytoplankton. The thoracic groove was visibly filled with phytoplankton before being brushed towards the mouthparts where the bolus continued to increase in size, before being rejected posteriorly. The formation and rejection of boluses indicate a krill’s inability to process phytoplankton efficiently when concentrations are so extreme. While such levels exceed typical open-ocean conditions, dense summer blooms in the Southern Ocean can approach similar values [9,10].

The physical variability in boluses was reflected in the differences in diet. Bolus size and consistency were influenced by phytoplankton cell size, shape and concentration. Although there was no mention of boluses, previous studies have suggested a reduced sieving efficiency when krill filter particles <10 µm [8,21,22]. Both flagellates from the present study were near this size limit, with *G. cryophila* (approx. 14 µm) being slightly larger than *P. gelidicola* (approx. 10 µm). The smaller cells congested the feeding basket, as *P. gelidicola* often resulted in the largest and densest boluses. In contrast, larger species *P. tricornutum* (approx. 30 µm), *C. flexuosus* (approx. 30 µm), *F. cylindrus* (approx. 80 µm) and *P. inermis* (approx. 200 µm) formed boluses that were rejected in the first 5 min, likely because their size allowed easier handling by the mandibles.

Handling time also influenced bolus compactness as those that were rejected earlier were visually less dense than those that remained within the basket for multiple compressions. The opposite is seen in faecal pellets where a longer absorption time results in less dense pellets over those produced by superfluous feeding, which results in denser pellets [23]. Bolus handling time may also be influenced by the cell shape. For example, physical factors such as spines or the formation of chains could allow mandibles to manipulate cells. Krill easily and quickly formed and rejected boluses from species such as *P. inermis*, a spiny diatom, and *F. cylindrus,* a chain-forming diatom.

Krill influence their surrounding environments by exporting an estimated 0.04 Gt C y^−1^ through faecal pellets in the marginal ice zone alone [24] and shift phytoplankton community structures through grazing [25]. Our results suggest that bolus production could aid in either event, contributing to carbon flux or recycling nutrients in surface layers. Larger, compact boluses, such as those made from *P. gelidicola*, sank considerably fast. This species was often retained in the feeding basket, resulting in visually denser and proportionally larger boluses reaching velocities up to 1403 m d^−1^. In contrast, smaller and visually less dense boluses were formed and rejected when krill fed on diatoms. This caused slower sinking velocities, as in *P. tricornutum,* an interesting observation considering diatoms provide opal (SiO_2_) as a ballasting material. This is consistent with observations that the ‘fluffiness’ of phytoplankton aggregates decreases sinking velocities [26,27]. *In situ*, these slower sinking particles may be more likely to be consumed in the surface layers, like other slow sinking aggregates and faecal pellets [28,29], while faster sinking boluses may transfer carbon more efficiently to depth. Additionally, the physical fragmentation of cells could lead to the recycling of nutrients, as has been observed with the rapid release of dissolved organic material when zooplankton perform sloppy feeding [30].

Furthermore, as other euphausiids also filter feed, bolus production is likely not unique to Antarctic krill. Indeed, previous observations of boluses within feeding baskets of Arctic krill [15] suggest that bolus formation may be a wide spread phenomenon amongst krill species.

Finally, increased bolus formation and rejection occurred when particles larger than phytoplankton cells were caught within the feeding basket. Faecal pellets clearly influenced bolus rejection and size. When krill were separated from the pellets with a grate to separate, the frequency of large boluses decreased (A. Butterley 2021, pers. obs.). Additionally, in an incidental case where equipment was contaminated with blue plastic (introduced via a cleaning sponge), bolus production increased approximately threefold (electronic supplementary material, table S4). In this instance, it was also found that thread or blue plastics were present in all rejected boluses. It is conceivable that krill sense and sort out less valuable food before ingesting the more valuable food items, but observations suggest that krill rejected these items due to an inability to tear them apart with their mandibular palps. These contaminated trials were excluded from all quantitative analysis, but the qualitative observation is noteworthy as these foreign materials influence the formation of boluses, acting as a seed and trapping phytoplankton when caught within the basket. Studies show that krill ingest artificial articles such as microplastics (<5 mm) [31], suggesting some may be ingested, while other larger particles may influence the formation of boluses. With rising microplastic pollution [32], this poses a potential concern, as krill may discard a high portion of their food if valuable particles stick to indigestible material.

## Data Availability

All data presented in this manuscript are available from the Dryad Digital Repository [33]. Supplementary material is available online [34].
